# Performance of Force-Field- and Machine Learning-Based Scoring Functions in Ranking MAO-B Protein–Inhibitor Complexes in Relevance to Developing Parkinson’s Therapeutics

**DOI:** 10.3390/ijms21207648

**Published:** 2020-10-16

**Authors:** Natarajan Arul Murugan, Charuvaka Muvva, Chitra Jeyarajpandian, Jeyaraman Jeyakanthan, Venkatesan Subramanian

**Affiliations:** 1 Department of Theoretical Chemistry and Biology, School of Chemistry, Biotechnology and Health, KTH Royal Institute of Technology, 10691 Stockholm, Sweden; 2 BiomAILS India Pvt Ltd., Hyderabad 500 090, India; charvakmuvva@gmail.com; 3 Department of Biotechnology, Dr. Umayal Ramanathan College for Women, Karaikudi 630 004, India; chitrajeyarajpandian@gmail.com; 4 Department of Bioinformatics, Alagappa University, Karaikudi 630 004, India; jjkanthan@gmail.com; 5 Centre for High Computing, CSIR-Central Leather Research Institute, Adyar, Chennai 600 020, India; subbu@clri.res.in

**Keywords:** binding free energy calculations, molecular docking, monoamine oxidase B, Parkinson’s disease, machine learning approach

## Abstract

Monoamine oxidase B (MAOB) is expressed in the mitochondrial membrane and has a key role in degrading various neurologically active amines such as benzylamine, phenethylamine and dopamine with the help of Flavin adenine dinucleotide (FAD) cofactor. The Parkinson’s disease associated symptoms can be treated using inhibitors of MAO-B as the dopamine degradation can be reduced. Currently, many inhibitors are available having micromolar to nanomolar binding affinities. However, still there is demand for compounds with superior binding affinity and binding specificity with favorable pharmacokinetic properties for treating Parkinson’s disease and computational screening methods can be majorly recruited for this. However, the accuracy of currently available force-field methods for ranking the inhibitors or lead drug-like compounds should be improved and novel methods for screening compounds need to be developed. We studied the performance of various force-field-based methods and data driven approaches in ranking about 3753 compounds having activity against the MAO-B target. The binding affinities computed using autodock and autodock-vina are shown to be non-reliable. The force-field-based MM-GBSA also under-performs. However, certain machine learning approaches, in particular KNN, are found to be superior, and we propose KNN as the most reliable approach for ranking the complexes to reasonable accuracy. Furthermore, all the employed machine learning approaches are also computationally less demanding.

## 1. Introduction

Parkinson’s disease (PD) is the second common progressive neurodegenerative disorder next to Alzheimer’s disease and it affects about 2% or 5 % of the population above 65 or 85 years of age, respectively, with 5–20 cases per 1,00,000 population every year worldwide [1]. The pathological hallmark of Parkinson’s disease includes progressive loss of dopaminergic cells present in the substantia nigra pars compacta in brain. In addition, non-dopamine pathways such as serotonergic, cholinergic and noradrenergic pathways also play a key role in the pathomechanism of Parkinson’s disease [2]. This pathological condition results in the deficiency of striatal dopamine in caudate nucleus and putamen with cytosolic Lewy bodies characterized by aggregated α-synuclein [3]. Deficiency of striatal dopamine cause syndrome characterized by Bradykinesia, motor disturbances, resting tremor and rigidity [4]. Apart from substantia nigra, the aggregated α-synuclein is also present all over the brain including autonomic nervous system that is connected to brain through vagal nerve [5]. There are several stages (1–6) in the progression of α-synucleinopathy. Stage 1 involves occurrence of aggregated α-synuclein in dorsal motor nucleus of vagal nerve and glossopharyngeal nerve [6]. Stage 2 involves extension of α-synucleinopathy to pontine tegmentum, locus coeruleus and medulla oblongata [6]. Stage 3 is characterized by loss of dopaminergic cells in substantia nigra that also affects amygdala region of the brain [6]. Stage 4 manifests marked degeneration of dopaminogenic neural cells of substantia nigra, Lewy pathology in temporal cortex and cardinal motor symptoms. Later, during stages 5 and 6, neurites and Lewy bodies occur in neocortex that results in cognitive impairment as in advanced stages of PD. Therefore, symptomatic phases precede the symptoms of movement disorder such as disturbance to the sensation of smell, rapid-eye movement in sleep, behavior disorder, depression, constipation and other cognitive symptoms [7]. Mitochondrial enzyme monoamine oxidase (MAO) exists in two isoforms MAO-A and MAO-B which are associated with degradation of various monoamines including dopamine, serotonin, epinephrine, norepinephrine, tyramine, phenylethylamine and benzylamine. MAO-A and MAO-B are equally active to dopamine and tyramine [8]. However, MAO-A shows higher activity towards deamination of serotonin and norephinephrin while MAO-B had greater activity to phenylethylamine and benzylamine [8]. Increased expression of MAO-B in brain due to aging and a specific allele of MAO-B gene in X chromosome are associated with the onset and progression of PD [8]. Based on the substrate, the MAO-B catalysis can proceed either by ternary or binary complex pathway. For instance, benzylamines are oxidized by ternary pathway while phenylethylamines are oxidized by binary or ping-pong pathway due to variation in the release of protonated imine relative to the oxidation rate of falvin–imine complex. In the case of benzylamine as substrate, the oxidation rate of imine complex is rapid than imine dissociation rate while the opposite is true for phenylethylamine substrate [8].

MAO-A is the major isoform in the non-central nervous system organs and therefore MAO-A deaminates tyramine in the gut. MAO-A inhibitors prevent deamination of tyramine in gut, but it is received by norepinephrine nerve terminals, and thereby it acts as false neurotransmitter by displacing stored norepinephrine [9]. This release of norepinephrine creates a hypertensive crisis called ‘cheese effect’. However, MAO-B does not react with tyrosine in the gut and therefore the MAO-B inhibitors are used in PD therapy without the need for low-tyramine diet restrictions [9]. Although both MAO-A and MAO-B are found in substantia nigra, MAO-A is mainly present in pars compacta while MAO-B is predominant in pars reticulate. Dietary tyrosine is first converted to levodopa in neurons which is decarboxylated to dopamine in both noradrenergic and dopaminergic neurons [10]. After dopamine is released from the synaptic vesicles, the MAO-B located in the glial cells around the synaptic cleft metabolizes dopamine [10]. Therefore, MAO-B inhibitors can be useful in the treatment of PD because they potentiate striatal response to dopamine which is the underlying pathology for dopamine deficiency. MAO-B are flavin adenine dinucleotide (FAD)-dependent enzymes found on the outer mitochondrial membrane of cells in both central nervous system and peripheral tissues [11].

**Figure 1:**
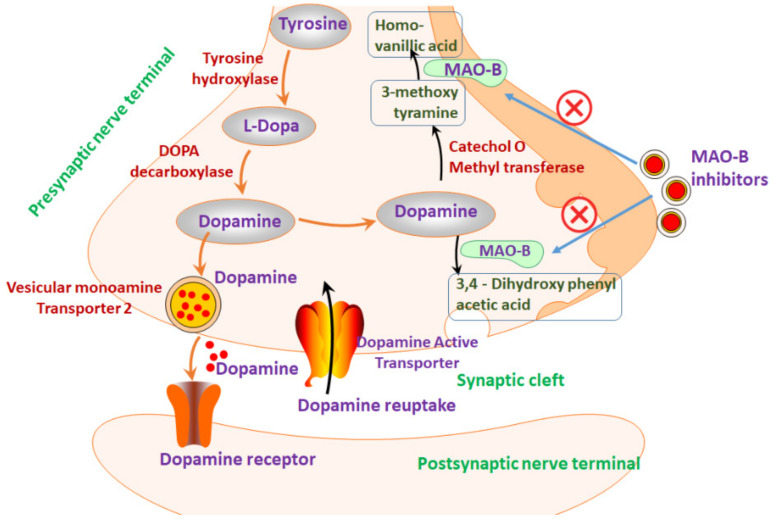
Mechanism of MAO-B inhibitors in PD.

MAO-B exists in dimeric form and each monomer unit has large globular structure (1–488 amino acid residues) exposed to solvent and a short helical domain (489–520 residues) in C-terminal bound to the lipid bilayer of mitochondrial membrane. The solvent exposed globular domain consists of both substrate-binding domain and FAD-binding domain [11]. A large cavity of about 700 Å${}^{3}$ in the substrate binding domain serves as the active site for the enzyme, and it opens on one side and extends further deep into the core until it extends to the inner face of the cofactor, FAD. About 461–488 residues form an extended loop from FAD-binding domain of MAO-B and connect the core protein to the C-terminal helix [12]. The active site of MAO-B is situated inside the substrate binding domain in each monomeric unit and contains two solvent inaccessible cavities. Substrates need to pass through the entrance cavity that opens to membrane bound side of MAO-B before binding to substrate cavity [12]. Both the substrate binding and entrance cavities are lined with hydrophobic aliphatic and aromatic residues. The substrate cavity in MAO-B is ellipsoidal disk shaped, which restricts the orientation of the inhibitor or substrate during binding so that the oxidized carbon atom binds to highly conserved regions near Flavin N5-C4 locus [12]. Crystallization studies of active site of MAO-B enzymes evidenced the presence of network of water molecules [13]. The cofactor FAD is bound to Cys-397 residue in C-terminal of MAO-B enzyme through covalent thioester linkage along with many other non-covalent interactions [13]. Hydrophobic interactions surround FAD within MAO-B and hydrogen bond interactions dominate bonding to both peptide bonds and amino acid side chains. Bond between positively charged Arg42 guanidine group and anionic pyrophosphate of FAD is the only electrostatic interaction exists between FAD and MAO-B [13]. Moreover, the Glu 34 carboxylate group is hydrogen bonded with the ribose group in the adenosine moiety of FAD and is essential for maintenance of structural integrity and covalent bonding of FAD within MAO-B [14]. Adenosine ribose group in FAD binds both with guanidine group of Arg 36 and water molecule [14]. These data evidence that extensive folding of enzyme MAO-B occurs to favor the interaction with FAD which is essential for the functional enzyme. MAO-B catalyzes the formation of 3,4-dihydroxyphenyl acetic acid and homovanillic acid dopamine by breakdown of dopamine [15]. These reactive metabolites of dopamine cause oxidative stress and dysfunction of mitochondria especially in complex 1 of electron transport chain [15]. Moreover, MAO-B metabolizes both exogenous and endogenous dopamine to form hydrogen peroxide, which is responsible for oxidative stress and damage in pathomechanism of PD [16]. Besides, MAO-B catalyzes breakdown of 1-methyl-4-phenyl 1,2,3,6-tetrahydropyridine (MPTP) to 1-methyl-4-phenylpyridinium ion (MPP+), which is a neurotoxic metabolite that can induce Parkinsonism in experimental animal models [16]. MAO-B can also induce catalytic activation of other neurotoxic metabolites such as β-carbolines and isoquinolines [16]. Therefore, inhibition of MAO-B activity by suitable MAO-B inhibitors could result in enhanced striatal dopaminergic activity with symptomatic benefit in dopamine deficit conditions. This could increase the amount and availability of dopamine to the dopamine receptors that consequently enhances the effects of levodopa. Moreover, MAO-B inhibitors reduce the free radicals generation due to dopamine oxidation and also inhibits the formation of MPP+ from MPTP in animal studies [17]. Therefore, the MAO-B inhibition could have neuroprotective and disease-modifying effects on PD. The mechanism of action of MAO-B inhibitors in PD is given in Figure 1. The MAO-B inhibitors increases the neuroprotective effects in PD when treated in combination with l-3,4-dihydroxyphenylalanine (l-DOPA- dopamine precursor) [18]. Therefore, several factors contribute to the efficacy of PD therapy including both non-pharmacological and pharmacological treatments. Until now, there is no definitive treatment for PD. Only few MAO-B inhibitors are approved for PD therapy and these drugs aid to recover from the neuropsychiatric and cognitive impairments in PD.

The effective therapy for the motor symptoms in PD includes modulation of dopamine system by dopamine agonists, exogenous dopamine and interfering the dopamine catabolism using monoamine oxidase (MAO-B) inhibitors. MAO-B inhibitors are commonly used for management of both psychiatric disorders and PD. Hence, development of potent MAO-B inhibitors, efficient delivery systems and novel combination drugs are of utmost importance due to the growing prevalence of PD. Given the above considerations, the present study explored the characteristics of established and experimental MAO-B inhibitors used in PD therapy. The objective of the study is to predict the binding affinities of bioactive compounds or drugs retrieved from ChEMBL database [19] against the MAO-B targets in PD by molecular docking, molecular mechanics energies combined with generalized Born and surface area continuum solvation (MM/GBSA) methods and regression analysis using machine learning approaches. The present study also identified the compounds with larger binding affinities as predicted using each of the force-field approaches. This study also discussed the most reliable method for predicting novel therapeutics for PD.

## 2. Results

**Figure 2:**
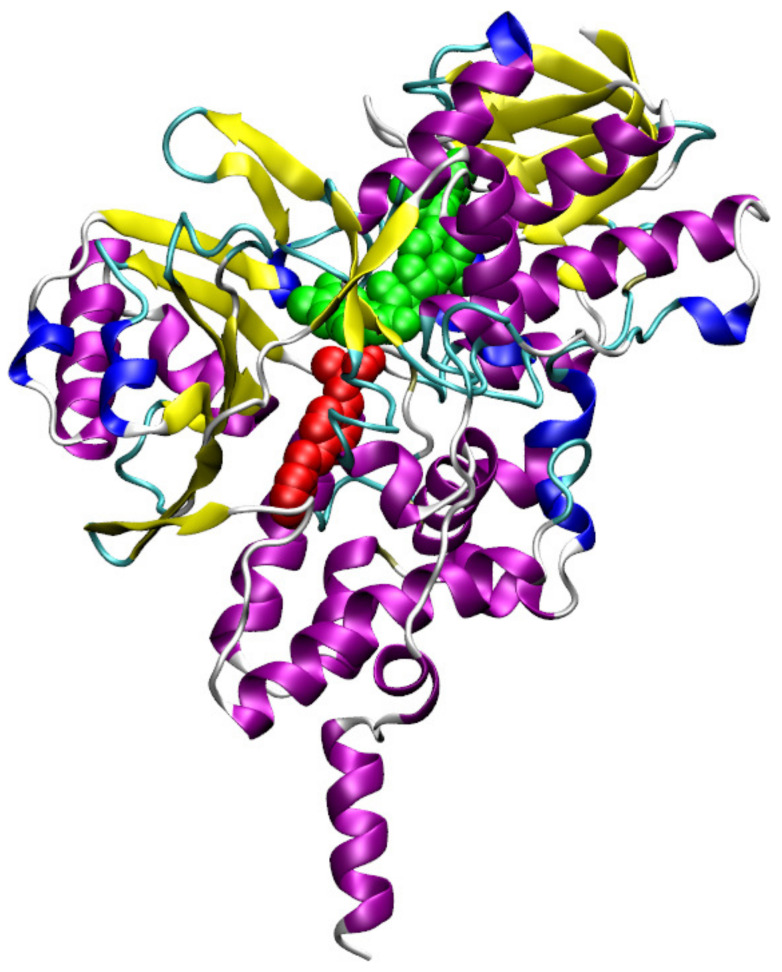
MAO-B safinamide complex as reported in the crystal structure (PDB id is 2v5z).

The structure of MAO-B enzyme in its safinamide bound state is displayed in Figure 2 which is based on the crystal structure reported in protein databank (PDB) with ID 2v5z [20]. The safinamide ligand is shown in red color using a VDW van der Waals) model and the co-factor FAD is in green color. The current study included docking of 3753 active compounds of human MAO-B from ChEMBL database [19]. The docking studies included the FAD as it is in the crystal structure. The experimental inhibition constants of this dataset of compounds are in the range from 59 mM to 0.01 nM. The compound 3-Methyl-8-(4,4,4-trifluoro-butoxy)-indeno[1,2-c]pyridazin-5-one with ID CHEMBL348961 is reported to have superior binding affinity (IC${}_{50}$ of 0.014 nM) while the compound 3-Benzylchroman-4-one with ID CHEMBL1766622 is the one with least binding affinity (IC${}_{50}$ of 58.8 mM). The SMILES information for top high affinity compounds along with their binding free energies are provided in Table S1. The chemical structure for CHEMBL348961 is given in Figure S1.

**Figure 3:**
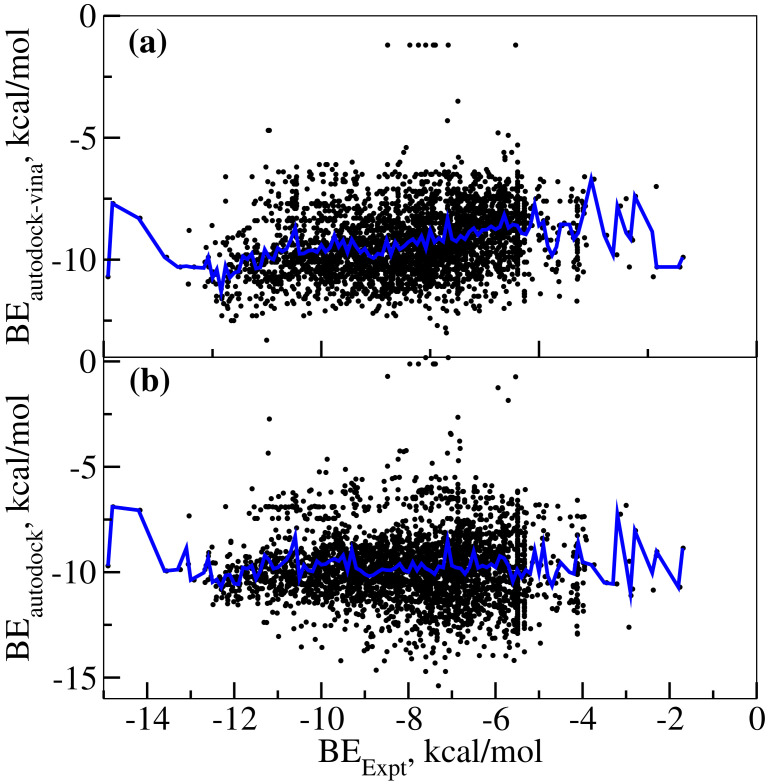
(**a**) Comparing the experimental and autodock-vina-based predicted binding free energies for various MAO-B ligand complexes. (**b**) Comparing the experimental and autodock4.0-based predicted binding free energies.The black spots represent different data points of the scatter diagram of experimental and predicted binding free energies. The plot in blue color shows F(BE${}_{expt}$) as a function of experimental binding free energies. For an excellent prediction, this plot should be the same as the expression, y = x.

Firstly, we analyzed the binding free energies predicted using autodock-vina software [21] and autodock [22] and only the least binding energies among the 10 binding modes for each ligand were used for ranking the complexes. In certain cases, the molecular docking did not get completed due to the mismatch between the ligand size and binding site cavity volume and these cases were carefully removed from the analysis. Figure 3a shows the plot of scatter diagram for predicted binding free energies using autodock-vina software and experimental binding free energies as we obtained from the ChEMBL database [19]. The binding free energies from IC${}_{50}$ values were obtained using the expression shown below:

(1) $$\Delta G=RTln(IC_{50})$$

Here, we approximated that inhibition constants are approximately equal to IC${}_{50}$ values. It is worth noting that the IC${}_{50}$ values are concentration dependent and so this approximation is valid only when the enzyme concentration is quite low. However, the IC${}_{50}$ values are proportional to binding affinities and hence can be directly compared to binding free energies estimated using different methods. A similar approximation has been followed by other researchers and IC${}_{50}$ values were used for computing the binding free energies of protein–ligand complexes [23,24]. In particular, the latter reference proves that the relative binding free energies of two ligands, A and B, are only proportional to their difference in IC${}_{50}$s ($\Delta G_{A,B}=RTlnIC_{50}A-RTlnIC_{50}B$) given that the target is the same as in the present case.

Using the above equation, the calculated binding free energies are in the range of −1.7 to −14.9 kcal/mol and the extreme values correspond to the compounds CHEMBL1766622 and CHEMBL348961, respectively. Now, we discuss the performance of molecular docking-based scoring in ranking the MAO-B:ligand complexes. Figure 3a is the scatter diagram of experimental binding free energies and predicted binding free energies. In addition, the average values of predicted free energies for different values of experimental free energies were computed and shown in the same plot as F(BE${}_{expt}$). This function relates the correlation between the experimental and binding free energies and computed from the averages of predicted binding free energies for different values of experimental binding free energies. For an excellent prediction, the plot of this function should be same as a plot for the expression, y = x. However, as we see in Figure 3a, the plot appears very much deviated from the ideal expression, y = x but rather looks closer to y = constant. The same is the case for the molecular docking results based on the autodock software, as shown in Figure 3b. The results clearly indicate that both methods fail badly in ranking the MAO-B: ligand complexes. Larger fluctuations are seen in the F(BE${}_{expt}$) for extreme values in BE${}_{expt}$ (i.e., for values of BE${}_{expt}$ below −13.0 and above −4 kcal/mol), which has to be attributed to the reduced number of datapoints available for this range of experimental binding energies. In line with our findings, the molecular docking approach has seen some criticism for its reliability in the literature [25]. However, we cannot ignore that the molecular docking approach has been successfully used to identify lead-drug-like compounds for various targets previously [26]. The current study is limited to MAO-B target and does not invalidate the use of molecular docking for screening of compounds.

**Figure 4:**
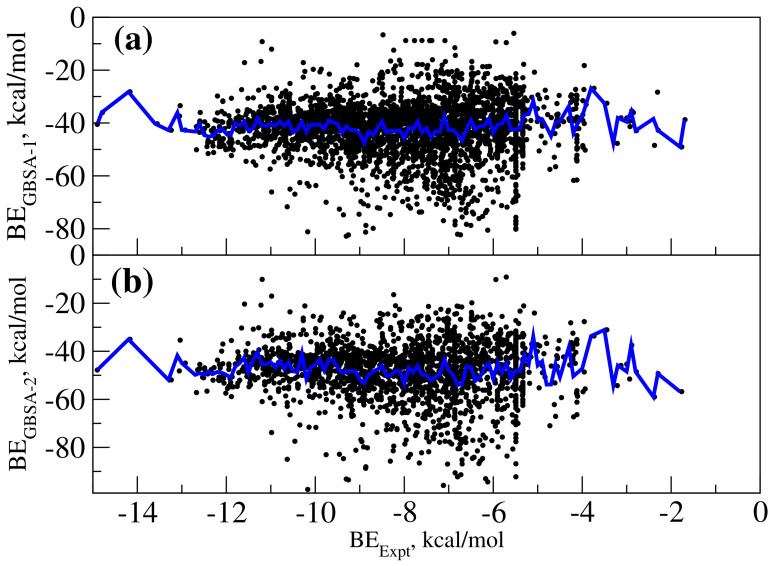
(**a**) Comparing the experimental binding free energies and MM-GBSA-based predicted free energies for various MAO-B ligand complexes. (**b**) Comparing the experimental and autodock-based predicted binding free energies. The black spots represent different data points of the scatter diagram of experimental and predicted binding free energies. The plot in blue color shows F(BE${}_{expt}$) as a function of experimental binding free energies.

We also computed the binding free energies using MM-GBSA approach and compared to the experimental binding affinities. Usually, the protein–ligand input structure used for computing MM-GBSA-based binding free energies can be obtained using different approaches. One can use the crystal structure or the structure from molecular docking studies using different softwares such as autodock-vina, autodock4.0 or GLIDE. Here, we carried out force-field molecular dynamics using the protein–ligand complex structures as obtained from docking study using autodock-vina and we computed two sets of MM-GBSA free energies. Set 1 uses the minimum energy structure for the complex as the input configuration. Set 2 uses as many as 100 configurations from molecular dynamics simulations carried out at 30 K. One can as well use the trajectories corresponding to ambient temperature simulations but then it is necessary to include entropic contributions which is computationally very demanding to estimate. In the low temperature simulations, the entropic contributions are negligible and so the binding enthalpies can be approximated directly for binding free energies. Figure 4a,b shows the correlation between the calculated binding free energies from Set 1 (referred to as GBSA-1 in Figure 4a) and Set 2 (referred to as GBSA-2 in Figure 4b) MM-GBSA approaches and experimental binding free energies respectively. It can be deduced that even these approaches are not able to rank the complexes correctly and there is no linear relationship observed between the experimental and computed binding free energies. The MM-GBSA-based binding free energies are observed to be quantitatively much lower when compared to autodock and autodock-vina-based binding free energies. It is very striking that our study shows that the MM-GBSA approach based on free energies cannot be used to compute the binding free energies of different MAO-B: ligand complexes.

**Figure 5:**
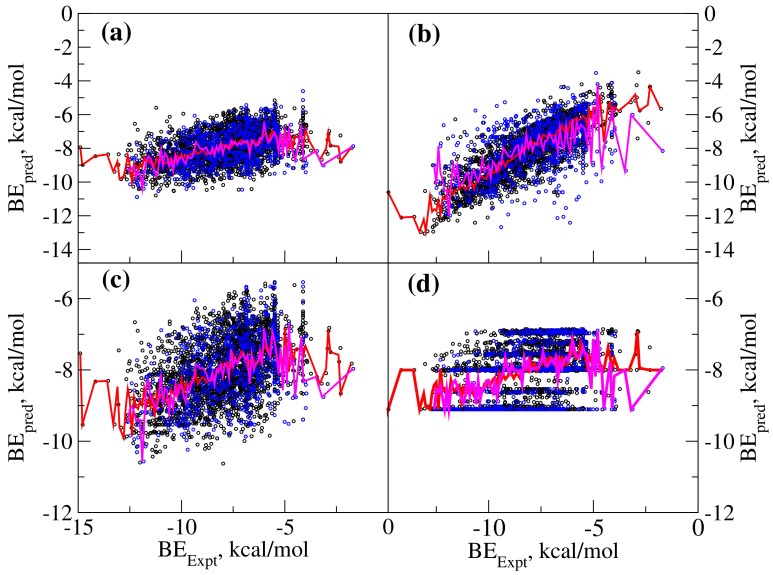
Comparing the experimental and machine learning-based predicted binding free energies for various MAO-B ligand complexes. The results from selected machine learning approaches: (**a**) logistic regression predictor; (**b**) k-nearest neighbor regressor; (**c**) multilinear perceptron regressor; and (**d**) random forest regressor. Black and blue dots refer to scatter diagram of experimental and predicted binding free energies and correspond to the results from training and test datasets, respectively. The plots in red and magenta color refer to F(BE${}_{expt}$) as a function of experimental binding free energies computed using training and test datasets, respectively.

Finally, we aimed to test the performance of data driven approaches which are becoming very popular recently for predicting various drug-like properties including the binding affinities. In particular, we employed supervised machine learning approaches and our dataset included various physicochemical properties computed for ligands as the descriptors and the binding free energy as the quantity to be predicted. The dataset was randomly divided into training (80% of the ligands included) and test (20% of the ligands included) datasets while all 3753 ligands make the full dataset. Different machine learning approaches were employed to predict the binding affinities for both the datasets. The ligands from the test dataset were not exposed to the machine learning models and so the correlation between the experimental and predicted binding free energies was expected to be lower when compared to that in the case of training dataset. We employed eight different machine learning approaches and four of the them performed better when compared to the rest. The results of those ML approaches performed better are shown in Figure 5a–d, which, respectively, correspond to: (a) linear regressor; (b) k-nearest neighbor regressor; (c) multilinear perceptron regressor; and (d) random forest regressor. Among these k-nearest neighbor algorithm is superior when compared to remaining three approaches. The root mean square deviation (RMSD) values between the experimental and predicted binding free energies for the training and test datasets using k-nearest neighbor algorithm are, respectively, 1.00 and 1.69. It is also worth recalling that these regressors only use the ligand information and so belongs to ligand-based approach while the above discussed force-field methods belong to structure-based approaches as they also include protein three dimensional structure information. Even then, the machine learning approaches were shown to be efficient in ranking the ligands. It may also be interesting to check the performance of machine learning approaches where the structural information of both protein and ligand are used to compute various descriptors [27]. However, we did not consider this in the current study.

## 3. Discussion

### 3.1. Force-Field Based Scoring Functions

Currently, there exist various force-field-based scoring functions to rank the protein–ligand complexes. In this study, we explored the performance of scoring functions as implemented in autodock-vina [21], autodock [22] and MM-GBSA [28]. In all approaches, the binding free energies are based on certain force-fields and are used for ranking the complexes. The lower is the value of binding free energies, the more stable the complex is suggested to be. The binding free energies in autodock and autodock-vina include the contributions from van der Waals, electrostatic, hydrogen bonding and solvation energies [29]. In addition, the entropic contributions associated with torsional degrees of freedom are also included. Each rotatable bond contributes with 0.2–0.3 kcal/mol to the entropy. The binding modes and binding poses with the least binding free energies for a given ligand within a target binding site are identified and included in the analysis. Usually, the protein framework is kept rigid, while the sampling over ligand translational, rotational and torsional degrees of freedom is carried out using Monte Carlo or Genetic Algorithm-based search approaches to identify the least energy binding modes. The most stable complex is the one associated with the least binding free energy, and this can be used as a scoring function for ranking this complex. The binding free energy calculations using MM-GBSA approach also include the contributions from van der Waals, electrostatic, polar and non-polar solvation free energies [28]. In this approach, solvation free energies are computed using an implicit solvent model [28]. The polar solvation energies are computed using the Generalized Born approach while the non-polar solvation free energies are computed using solvent accessible surface area. The entropic contributions due to vibrational degrees of freedom can also be estimated by computing normal modes. In most cases, the binding enthalpies are treated as binding free energies and the entropic contributions are assumed to be negligible factors in ranking the protein–ligand complexes. There have been many success stories reported on the ranking of complexes using force-field-based scoring functions [30]. However, here, all three methods (autodock-vina, autodock and MM-GBSA) fail in ranking the complexes to any reasonable accuracy. The Supplementary Materials provide the SMILES of top high affinity compounds predicted from each of these four approaches. Tables S2–S5, respectively, correspond to the results from autodock-vina, autodock and MM-GBSA Set 1 and Set 2. In addition, the Supplementary Materials provide the chemical structure of the topmost high affinity compound from each of these approaches.

The machine learning-based approaches can be used for predicting any properties given that there exists suitable dataset which correlates the property to be predicted to set of molecular descriptors. In this study, we used dataset of SMILES and IC${}_{50}$ for 3753 ligands available in ChEMBL database. From SMILES, one can compute various descriptors such as physicochemical properties, rule of 5, various molecular finger prints and the dataset of all these or selected descriptors along with the property (IC${}_{50}$ here) can be used to train a machine learning model which can be used to predict the property for a given new sets of descriptors or a SMILES datum. In this particular study, only the physicochemical properties were used as the features to develop machine learning models. Further, the following machine algorithms [31,32,33,34,35,36,37,38] were used: Random Forest (RF), Decision Tree (DT), Multilayer-Perception (MLP), Support Vector Machine (SVM), Logistic Regression (LR), kappa Nearest Neighbor (kNN), Stochastic Gradient Descent (SGD) and Gaussian Naive Bayes (GNB). Among these, only four of the machine learning models, namely RF, kNN, MLP and LP, performed to a reasonable extent (as the relationship between the experimental and predicted binding free energies can be fitted to an expression, y = mx + c). Among these, kNN appears to be the best regressor for predicting the binding affinities as the correlation almost overlaps with the expression, y = x. In addition, the RMSD values which are quantitative measures to describe the performance of the model were found to be lower for the results from training and test datasets.

### 3.2. Multiple Binding Sites in MAO-B

**Figure 6:**
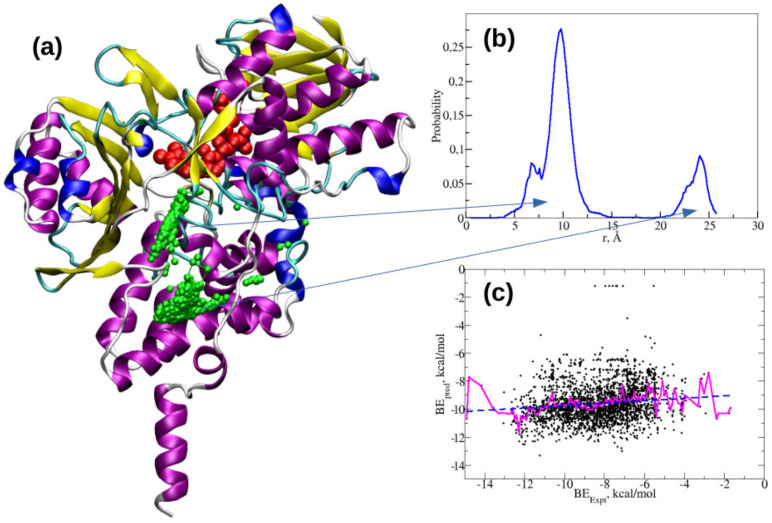
(**a**) Two major binding sites in MAO-B for various ligands; (**b**) the distribution of distances between the FAD-centered N atom and center of mass of ligands; and (**c**) the correlation between the autodock-vina predicted binding free energies and experimental binding free energies for the ligands in substrate cavity site.

The substrate cavity space located next to FAD cofactor is the most attractive binding site for the ligands (see Figure 2). There are structural reports available for various MAO-B–ligand complexes and they also suggest that this site is the most preferable site for binding [12,13,39,40]. Previous computational studies on tracers have also reported that these ligands bind to the same substrate binding site as the safinamide and pioglitazone [40,41,42]. In agreement to this, autodock-vina and autodock also suggest this as the high affinity binding site for a number of compounds. However, some of the compounds bind to entrance cavity while very few are found to bind with the so called imidazolium binding site (which is also located near the entrance cavity). Certain studies showed that when the substrate cavity space is occupied by the tranylcypromine-like irreversible inhibitors (others being rasagiline and deprenyl), the entrance cavity site is the most preferable site for the ligands [43,44]. We analyzed the binding sites for the ligands and found that many of the ligands are binding to substrate binding cavity (as many as 78.3%) and remaining ligands bind to entrance cavity site (see Figure 6a). The distribution of distance between the FAD-centered N atom and center of mass of the ligand shows a bimodal distribution indicating that these two sites are the most preferable binding sites in MAO-B (see Figure 6b). We also identified those ligands within the substrate binding site and only analyzed whether the autodock-vina predicted docking energies correlate with experimental binding affinities. The results are shown in Figure 6c, but, as can be seen, the correlation between these two properties is still not very impressive.

## 4. Materials and Methods

### 4.1. Binding Affinity Data-Set Retrieval and Preprocessing

We searched the ChEMBL database [19] for the target human monoamine oxidase B. There were more than 5000 molecules, peptides and biologics showing activity against the target. We downloaded all the compounds and filtered for small molecules (by setting the compound molecular weight to be lower than 500 Da). The compounds in the salt forms were identified and the counter-ions were removed. Further, the compounds having IC${}_{50}$ values were filtered out separately and used in this study. The number of compounds after the filtering was 3753, and we computed different scoring functions (both force-field-based and machine learning-based) for these compounds as described below. The SMILES were converted to mol2 files using rdkit tools [45]. Initially, optimization using PM7 semiempirical level of theory and the geometry optimized structures were used for the molecular docking using autodock-vina and autodock.

### 4.2. Molecular Docking and MM-GBSA Based Binding Free Energy Calculations

The geometry optimized structures for the 3753 compounds were used for the molecular docking using autodock-vina [21] and autodock [22]. These calculations involve the following steps: The gaussian output files were converted to mol2 files which are then converted to pdbqt files using openbabel software [46]. The three-dimensional structure for MAO-B target used in the molecular docking studies was based on the structure with PDB id 5v2z, as reported in the protein databank [47]. The safinamide inhibitor bound to the target was removed but the FAD was kept intact in the molecular docking study. We chose the substrate cavity site as the binding site for docking. The grid box dimensions were chosen as 30 × 30 × 30 with a default grid spacing of 0.375 Å. The 10 lowest energy binding modes were computed using the two molecular docking softwares (autodock-vina and autodock). The least energy binding mode and the corresponding binding free energies were collected for further analysis and for studying the performance of the scoring functions based on the force-fields as implemented in molecular docking software.

The most stable binding modes from molecular docking calculations using autodock-vina software were used for generating the input configuration for molecular dynamics and MM-GBSA calculations. The molecular dynamics calculations require the charges and force-fields for the ligands. Thus, the molecular structure as in the most stable binding mode for each of ligands was used for generating electrostatic potential fitted charges using Merz–Kollman–Singh scheme [48,49]. Density functional theory with B3LYP correlation exchange functional and 6-31+G(d) basis sets [50,51,52] as implemented in Gaussian09 software [53] was employed for this calculation. Furthermore, general amber force-field (GAFF) [54] was used for describing the van der Waals interactions in the molecular dynamics study. The ESP charges computed using Merz–Kollman–Singh scheme and GAFF force-field were used for describing the FAD cofactor as well. For describing the MAO-B protein, FF99SB force-field was used. The FAD cofactor is in a dianionic state while the protein is charged with −3. Thus, sufficient numbers of counter ions and water solvents were added to prepare the solvated complex structure for carrying out molecular dynamics simulations. A minimization run was carried out for all the complexes and a short simulation in isothermal-isobaric ensemble was carried out. In particular, the finite temperature simulations were carried out at 30 K and 1 atm pressure. The final configuration from minimization run and 100 configurations at finite temperature simulations were used for computing two sets of binding free energies, respectively, using MM-GBSA approach. These binding free energies were further used to rank all MAO-B:ligand complexes.

### 4.3. Descriptors Calculation and Machine Learning Model Building

In total, 3753 biologically active compounds for the target, human MAO-B, were selected for machine learning calculations. The physicochemical descriptors were used to predict the biological activity. For physicochemical descriptors, 1054 molecular descriptors, including Acidic Group Count, ALOGP, APol, Aromatic Atoms Count, Aromatic Bonds Count, Atom Count, Autocorrelation, Basic Group Count, Bond Count, Constitutional, Crippen, Detour Matrix, EState Atom Type, Extended Topochemical Atom, Fragment Complexity, HBond Acceptor Count, HBond Donor Count, Hybridization Ratio, Ring Count, Rotatable Bonds Count, Topological, Topological Charge, Topological Distance Matrix, TPSA, Weight, XLogP, etc., were calculated using PaDEL software [55]. It is known that the use of raw data in the machine learning would lead to artifacts (as certain descriptors having larger magnitudes will be over-described and will dominate the performance of the model). To avoid this, data were normalized. Further, to improve the computational efficiency and to reduce the generalization error of the model by removing irrelevant features or noise, the Sequential Feature Selection (SFS) algorithm was employed. Based on the performance score, the ATS5m, ATS8i, ATSC2v, ATSC5v, ATSC3i, Sp, SpAD_Dt, SpMAD_Dt, SdO, fragC features were selected to build the machine learning models. Eight different machine learning methods from the Scikit-learn module [45] and the open-source python3.8 package were used to predict the biological activity: Random Forest (RF), Decision Tree (DT), Multilayer-Perception (MLP), Support Vector Machine (SVM), Logistic Regression (LR), kappa Nearest Neighbor (kNN), Stochastic Gradient Descent (SGD), and Gaussian Naive Bayes (GNB) [31,32,33,34,35,36,37,38].

## 5. Conclusions

Parkinson’s disease is one of the most common neurodegenerative diseases and is associated with accumulation of alpha-synuclein fibrils. In addition, it is also associated with dopamine deprival which is essential for motor activities in humans. As this particular amine is degraded by MAO-B enzyme, its deprival can be controlled by using MAO-B inhibitors and thus these are considered for the treatment of PD associated symptoms. One can computationally use structure-based and ligand-based approaches to design various high affinity compounds for this target. The ChEMBL server provides a list of more than 3700 organic compounds with activity against this target and this dataset can be used for designing the compounds using data-driven approaches. We employed both force-field-based and data driven approaches to rank the complexes. All three force-field-based approaches cannot rank the complexes to any accuracy while the performance of certain machine learning approaches is very encouraging. In particular, the machine learning algorithm KNN outperforms all the methods tested in ranking the complexes. Given that the machine learning approaches are not very computationally demanding and they are also reliable in ranking the complexes, they can be used for screening compounds from various chemical spaces such as ZINC or Cambridge, towards a specific target for identifying lead drug-like compounds.

## Supplementary Materials

The following supplementary material for this article can be found online at https://www.mdpi.com/1422-0067/21/20/7648/s1.

## Abbreviations

The following abbreviations are used in this manuscript:

FAD	Flavin adenine dinucleotide
MAOB	Monoamine Oxidase B
ML	Machine learning
MM-GBSA	Molecular mechanics-Generalized Born surface Area approach
