# Supplementary material for: Performance of Force-Field- and Machine Learning-Based Scoring Functions in Ranking MAO-B Protein–Inhibitor Complexes in Relevance to Developing Parkinson’s Therapeutics
Source: Int J Mol Sci. 2020 Oct 16;21(20):7648. doi: 10.3390/ijms21207648 (PMC7589968; doi:10.3390/ijms21207648)
Supplement: Supplementary file 1 [file ijms-21-07648-s001.pdf]

Supporting information
for

Performance of Force-field and Machine Learning Based Scoring Functions in Ranking MAOB Protein-inhibitor Complexes in Relevance to Developing Parkinson's Therapeutics

Natarajan Arul Murugan[1], Charuvaka Muvva[2], Chitra Jeyarajpandian[3], Jeyaraman Jeyakanthan[4], Venkatesan Subramanian[5]

[1]Department of Theoretical Chemistry and Biology, School of Chemistry, Biotechnology and Health, KTH Royal Institute of Technology, Stockholm, Sweden;
Email : murugan@kth.se

[2]BioMAILS India Pvt Ltd, Hyderabad, India

[3]Department of Biotechnology, Dr. Umayal Ramanathan College for Women, Karaikudi, India

[4]Department of Bioinformatics, Alagappa University, Karaikudi, Tamilnadu, India

[5]Centre for High Computing, CSIR-Central Leather Research Institute, Adyar, Chennai – 600 020, India.

Table S1: List of high affinity compounds as reported from experiments. The binding free energies are in kcal/mol

-14.899	Cc1cc2c(nn1)-c1cc(OCCCC(F)(F)F)ccc1C2=O
-14.783	C#CCN(C)[C@H](C)Cc1ccccc1
-14.165	O=C(O)c1coc2ccccc2c1=O
-13.553	Cc1ccc2oc(=O)c(-c3cccc(Br)c3)cc2c1
-13.238	O=C(Nc1ccc(Cl)c(Cl)c1)c1ccc2[nH]ccc2c1
-13.056	Cc1ccc(-c2cc3cc(C)ccc3oc2=O)cc1
-13.037	O=C1CCc2cc(OCCCC(F)(F)F)ccc21
-12.922	Cn1ncc2cc(C(=O)Nc3ccc(Cl)c(Cl)c3)ccc21
-12.768	O=c1ccc2ccc(OCc3ccc(Br)cc3)cc2o1
-12.673	O=C(Nc1ccc(Cl)c(Cl)c1)c1ccc2[nH]ncc2c1
-12.671	COC(=O)c1cc2ccc(OCc3cccc(F)c3)cc2oc1=O
-12.647	Clc1ccc(/N=C/c2ccc3[nH]ncc3c2)cc1Cl
-12.602	O=C1Nc2ccc(CCCCc3ccccc3)cc2C1=O
-12.600	Cn1ncc2cc(C(=O)Nc3ccc(F)c(Cl)c3)ccc21
-12.595	O=C(Nc1ccc(Cl)c(F)c1)c1ccc2[nH]ncc2c1
-12.585	O=C(Nc1ccc(F)c(Cl)c1)c1ccc2[nH]ncc2c1
-12.534	COc1ccc(-c2cc3cc(C)ccc3oc2=O)cc1Br
-12.510	C#CCCCCOc1ccc2c(C)cc(=O)oc2c1
-12.487	COc1cccc(-c2cc3cc(C)ccc3oc2=O)c1
-12.487	Cc1c(Cl)c(=O)oc2cc(OCc3ccc(Br)cc3)ccc12
-12.485	COc1cccc(-c2cc3cc(C)ccc3oc2=O)c1
-12.434	CCCCCCOc1ccc2cc(C(=O)OC)c(=O)oc2c1
-12.417	Cc1cc(=O)oc2cc(OCc3ccc(Br)cc3)ccc12
-12.417	Cc1c(C)c2ccc(OCc3nnc(C(C)C)s3)cc2oc1=O
-12.409	O=c1oc2cc(OCc3ccccc3)ccc2c2c1CCC2
-12.409	Cc1c(C)c2ccc(OCc3cccc(F)c3)cc2oc1=O
-12.337	Cn1ncc2cc(/C=N/c3ccc(Cl)c(Cl)c3)ccc21
-12.313	Cc1c(C)c2ccc(OCc3ccccc3)cc2oc1=O
-12.309	COCCn1ncc2cc(C(=O)Nc3ccc(Cl)c(Cl)c3)ccc21
-12.300	Cc1c(C)c2ccc(OCc3cccc([N+](=O)[O-])c3)cc2oc1=O
-12.298	Cc1c(C)c2ccc(OCc3ccc(F)c(F)c3)cc2oc1=O
-12.286	Cc1c(C)c2ccc(OCc3ccc([N+](=O)[O-])cc3)cc2oc1=O
-12.286	O=c1oc2cc(OCc3ccccc3)ccc2c2ccccc12
-12.276	Cc1c(C)c2ccc(OCc3ccc(F)c(F)c3)cc2oc1=O
-12.256	C=C(Br)COc1ccc2c3c(c(=O)oc2c1C)CCC3
-12.245	Cc1cccc(COc2ccc3c(C)c(C)c(=O)oc3c2)c1
-12.198	CC(=O)c1ccc(OCc2cccc(Cl)c2)cc1O
-12.198	C#CCN(C)[C@H](C)Cc1ccccc1
-12.198	Cc1c(C#N)c(=O)oc2cc(OCc3ccc(Br)cc3)ccc12
-12.175	COc1ccc(-c2cc3cc(Br)cc(OC)c3oc2=O)cc1
-12.162	Cc1c(C)c2ccc(NCc3ccccc3)cc2oc1=O
-12.162	Cc1ccc(COc2ccc3c(C)c(C)c(=O)oc3c2)cc1
-12.154	O=C1OCc2ccc(OCc3ccc(C(F)(F)F)cc3)cc21
-12.154	CS(=O)(=O)c1ccc(NC(=O)c2cc3ccccc3oc2=O)cc1

Table S2: List of high affinity compounds as predicted from Autodock-vina. The binding free energies are in kcal/mol

-13.300	O=C1c2ccccc2-c2nnc(-c3ccc4ccccc4c3)cc21
-13.000	O=c1c(/C=N/c2cc3ccccc3cc2O)coc2ccccc12
-12.800	C#CCNC1CCc2c(OCc3ccc4ccccc4c3)cccc21
-12.700	Cc1ccc(NC(=O)c2coc3ccc(/C=C/C(=O)O)cc3c2=O)cc1C
-12.500	O=c1oc2cc(OCc3ccccc3)ccc2c2c1CCCC2
-12.500	Cc1c(C)c2ccc(OCc3c(F)c(F)c(F)c(F)c3F)cc2oc1=O
-12.400	O=c1oc2cc(/N=C/c3ccc(O)cc3O)ccc2c2c1CCCC2
-12.300	CC(C)c1ccc(NC(=O)c2cc3ccccc3oc2=O)cc1
-12.300	Cc1c(C)c2ccc(OCc3cccc(OC(F)(F)F)c3)cc2oc1=O
-12.300	O=c1oc2cc(OCc3ccccc3)ccc2c2ccccc12
-12.300	Cc1c(C)c2ccc(OCc3ccccc3C#N)cc2oc1=O
-12.300	Cc1c(C)c2ccc(OCc3cccc(C(F)(F)F)c3)cc2oc1=O
-12.300	O=C(Nc1ccccc1Cc1ccccc1)c1cc2ccccc2oc1=O
-12.300	O=C1c2ccccc2-c2nnc(/C=C/c3ccccc3)cc21
-12.200	O=c1oc2cc(/N=C/c3ccccc3O)ccc2c2c1CCCC2
-12.200	Cc1ccc(NC(=O)c2cc3ccccc3oc2=O)cc1C
-12.200	Cc1cn(-c2ccc3nc(-c4ccc5c(c4)OCO5)ncc3c2)cn1
-12.200	O=C1c2ccccc2-c2nnc(-c3ccc(C(F)(F)F)cc3)cc21
-12.200	O=C1NC(=O)c2cc(Oc3ccc4ccccc4c3)ccc21
-12.100	O=c1oc2cc(NCc3ccccc3O)ccc2c2c1CCC2
-12.100	C(=N/Nc1nc(-c2ccc3ccccc3c2)cs1)c1cccc2ccccc12
-12.100	Cc1ccc2oc(=O)c(-c3cccc(OCc4cccc(F)c4)c3)cc2c1
-12.100	Cn1c(=O)c2c(cc(/C=C/c3ccc(C(F)(F)F)cc3)n2C)n(C)c1=O
-12.100	Cc1ccc2oc(=O)c(-c3cccc4ccccc34)cc2c1
-12.000	O=C(Nc1c(F)c(F)c(F)c(F)c1F)c1cc2ccccc2oc1=O
-12.000	Cc1c(C)c2ccc(OCc3cccc(C#N)c3)cc2oc1=O
-12.000	O=C(Nc1cccc(C(F)(F)F)c1)c1cc2ccccc2oc1=O
-12.000	O=C1c2ccccc2-c2nnc(-c3ccc(C(F)(F)F)cc3)cc21
-12.000	O=C1c2ccccc2-c2nnc(-c3cccc(C(F)(F)F)c3)cc21
-12.000	C#CCN1CCCC(CNC(=O)c2ccc3ccccc3c2)C1
-12.000	Cc1ccc2oc(=O)c(-c3c[nH]c4ccccc34)cc2c1
-11.900	O=C(Nc1cccc(C(F)(F)F)c1)c1cc2ccccc2oc1=O
-11.900	Cc1c(C)c2ccc(OCc3ccc(C#N)cc3)cc2oc1=O
-11.900	O=C(Nc1cccc(C(F)(F)F)c1)c1cc2cc([N+](=O)[O-])ccc2oc1=O
-11.900	N#Cc1c(F)c(F)c(NC(=O)c2cc3ccccc3oc2=O)c(F)c1F
-11.900	Cc1cccc(NC(=O)c2cc3ccccc3oc2=O)c1C
-11.900	Cc1ccc(COc2ccc3c(C)c(C)c(=O)oc3c2)cc1
-11.900	O=C1COC(c2ccc(NC(=O)Nc3cccc(Cl)c3)cc2)=NN1
-11.900	Cc1cccc(NC(=O)c2cc3cc(C)ccc3oc2=O)c1
-11.900	Cn1c(=O)c2c(cc(/C=C/c3cccc(C(F)(F)F)c3)n2C)n(C)c1=O
-11.900	O=c1oc2cc(/N=C/c3ccccc3O)ccc2c2c1CCC2
-11.900	Cc1c(C)c2ccc(OCc3ccc(F)c(F)c3)cc2oc1=O
-11.800	O=C1CCCc2cc(OCc3ccc(C(F)(F)F)cc3)ccc21
-11.800	Cc1cccc(COc2ccc3c(C)c(C)c(=O)oc3c2)c1
-11.800	CCc1ccc(NC(=O)c2cc3ccccc3oc2=O)cc1
-11.800	Cc1ccc(NC(=O)c2cc3ccccc3oc2=O)c(C)c1

-11.800	Cc1cc(C)cc(NC(=O)c2cc3ccccc3oc2=O)c1
-11.800	O=C(Nc1ccc(C(F)(F)F)cc1)c1coc2ccccc2c1=O
-11.800	O=C1/C(=C/c2ccc(Cl)c(Cl)c2)Cc2cc(O)ccc21
-11.800	O=C(Nc1ccc(OC(F)(F)F)cc1)c1coc2ccccc2c1=O

Table S3: List of high affinity compounds as predicted from autodock4.0. The binding free energies are in kcal/mol

-15.390	Cc1c2c(nn1-c1ccc(/C=N/NC(=S)NCc3ccccc3)cc1)-c1ccccc1S(=O)(=O)N2C
-14.710	Cc1c2c(nn1-c1ccc(/C=N/NC(=S)Nc3ccccc3Cl)cc1)-c1ccccc1S(=O)(=O)N2C
-14.710	Cc1c2c(nn1-c1ccc(/C=N/NC(=S)Nc3cccc(Cl)c3)cc1)-c1ccccc1S(=O)(=O)N2C
-14.650	Cc1c2c(nn1-c1ccc(/C=N/NC(=S)Nc3ccc(Cl)cc3)cc1)-c1ccccc1S(=O)(=O)N2C
-14.580	Cc1ccc(NC(=S)N/N=C/c2ccc(-n3nc4c(c3C)N(C)S(=O)(=O)c3ccccc3-4)cc2)cc1
-14.310	Cc1c2c(nn1-c1ccc(/C=N/NC(=S)Nc3ccccc3)cc1)-c1ccccc1S(=O)(=O)N2C
-14.210	COc1ccccc1CN(C)CCCCOc1cc(O)c2c(=O)c3ccccc3sc2c1
-14.200	Cc1ccc(NC(=S)N/N=C/c2ccc(-n3nc4c(c3C)N(C)S(=O)(=O)c3ccccc3-4)cc2)c(C)c1
-14.190	O=C(CCCCCCNC(=O)c1ccc(CNC2(c3ccccc3)CC2)cc1)NO
-14.110	Cc1c2c(nn1-c1ccc(/C=N/NC(=S)Nc3cccc(F)c3)cc1)-c1ccccc1S(=O)(=O)N2C
-14.050	COc1ccccc1CN(C)CCCOc1cc(O)c2c(=O)c3ccccc3sc2c1
-14.020	CCc1ccc(NC(=S)N/N=C/c1ccc(-n2nc3c(c2-c2ccccc2)N(C)S(=O)(=O)c2ccccc2-3)cc1
-14.010	Cc1c2c(nn1-c1ccc(-c3ccc(Cl)nn3)c(=O)oc12
-13.970	O=C(Nc1ccccc1Cc1ccccc1)c1cc2ccc(OCc3ccccc3)cc2oc1=O
-13.960	C(=N/Nc1nc(-c2ccc3ccccc3c2)cs1)c1cccc2ccccc12
-13.950	O=C1/C(=C/c2ccc(OCCCN3CCN(Cc4ccccc4)CC3)cc2)COc2ccccc21
-13.900	CCCn1c(=O)c2c(nc3n2CCCN3CCC(=O)N2CCN(c3ccccc3)CC2)n(CCC)c1=O
-13.830	c1ccc2c(c1)c1cnccc1n2CCCCCCCn1c2ccccc2c2cnccc21
-13.830	CCN(CCCOc1cc(O)c2c(=O)c3ccccc3sc2c1)Cc1ccccc1N(C)C
-13.790	Cc1c2c(nn1-c1ccc(/C=N/NC(=S)Nc3ccccc3F)cc1)-c1ccccc1S(=O)(=O)N2C
-13.780	CS(=O)(=O)Nc1ccc(-c2coc3cc(C#Cc4cc[nH]c(=O)c4)ccc3c2=O)cc1
-13.770	CN1CCc2c(c3ccccc3n2CCCCCCCn2c3c(c4ccccc42)CN(C)CC3)C1
-13.740	CCN(CCCOc1cc(O)c2c(=O)c3ccccc3sc2c1)Cc1ccccc1
-13.730	CCN(CCCCOc1cc(O)c2c(=O)c3ccccc3sc2c1)Cc1ccccc1
-13.700	c1ccc2c(c1)c1cnccc1n2CCCCCCn1c2ccccc2c2cnccc21
-13.680	COc1ccc(/C=C2COc3cc(OCCCCCCNc4c5c(nc6cc(Cl)ccc46)CCCC5)ccc3C2=O)cc1
-13.640	Cc1ccc(S(=O)(=O)Oc2cccc(-c3cc4cc(C)ccc4oc3=O)c2)cc1
-13.590	c1ccc2c(c1)c1cnccc1n2CCCCCCCCCn1c2ccccc2c2cnccc21
-13.570	CN(C)c1ccc(-c2nnc3c(C(=O)c4ccccc4)nc4ccccc4n23)cc1Br
-13.570	Cc1c(C)c2ccc(OCc3ccc(CN(C)Cc4ccccc4)cc3)cc2oc1=O
-13.550	CN(Cc1ccc(COc2ccc3c(CO)cc(=O)oc3c2)cc1)Cc1cccc(Cl)c1
-13.550	CN1CCc2c(c3ccccc3n2CCCCCCn2c3c(c4ccccc42)CN(C)CC3)C1
-13.550	CCN(CCCCOc1cc(O)c2c(=O)c3ccccc3sc2c1)Cc1ccccc1N(C)C
-13.530	CN(CCCCOc1cc(O)c2c(=O)c3ccccc3sc2c1)Cc1ccccc1
-13.530	Cc1ccc(NC(=O)CCC(=O)OCC(=O)c2ccc(-c3ccccc3)cc2)cc1Cl
-13.490	O=c1oc2cc(OCCNC3CCN(Cc4ccccc4)CC3)ccc2c2c1CCCC2
-13.470	CCN(CCCCOc1cc(O)c2c(=O)c3ccccc3sc2c1)Cc1ccccc1OC
-13.460	Cn1c(CNc2ccccc2)cc2cc(OCCCC3CCN(Cc4ccccc4)CC3)ccc21
-13.390	C(=N/Nc1nc(-c2ccc3ccccc3c2)cs1)c1c[nH]c2ccccc12
-13.390	Cc1c(C#N)c(=O)oc2cc(OCCN3CCC(Cc4ccccc4)CC3)ccc12
-13.380	CCN(CCCOc1cc(O)c2c(=O)c3ccccc3sc2c1)Cc1ccccc1OC
-13.370	CC1(c2ccccc2)OC(/C=C/COc2ccc3ccc(=O)oc3c2)=CC1=O

-13.370	O=C(c1ccccc1)c1nc2ccccc2n2c(-c3ccccc3)nnc12
-13.360	CCN(CCCCCCOc1cc(O)c2c(=O)c3ccccc3sc2c1)Cc1ccccc1
-13.340	c1ccc2c(c1)c1cnccc1n2CCCCCCCCn1c2ccccc2c2cnccc21
-13.330	Clc1ccccc1-c1nnc2c(Cc3ccccc3)nc3ccccc3n12
-13.290	C#CCN(C)[C@H](C)Cc1ccc(OCCCNc2c3c(nc4cc(Cl)ccc24)CCCC3)cc1
-13.230	Cc1cc2ccc(OCCNC3CCN(Cc4ccccc4)CC3)cc2oc1=O
-13.230	C#CCN(C)[C@H](C)Cc1ccc(OCCCNc2c3c(nc4ccccc24)CCCC3)cc1
-13.230	N#Cc1ccc(-c2csc(N/N=Cc3cccc4ccccc34)n2)cc1

Table S4: List of high affinity componds as predicted from MM-GBSA set-1. The binding free energies are in kcal/mol

-82.816	C#CCN(C)[C@H](C)Cc1ccc(OCCCCCCCCNc2c3c(nc4ccccc24)CCCC3)cc1
-82.324	C#CCN(C)[C@H](C)Cc1ccc(OCCCCCCCCCNc2c3c(nc4ccccc24)CCCC3)cc1
-82.289	CCN(CCCCCCn1sc2cc(OC)c(OC)cc2c1=O)Cc1ccc(N(C)C)cc1
-82.065	C#CCN(C)[C@H](C)Cc1ccc(OCCCNc2c3c(nc4cc(Cl)ccc24)CCCC3)cc1
-81.278	C#CCN(C)[C@H](C)Cc1ccc(OCCCCCCCCCCNc2c3c(nc4ccccc24)CCCC3)cc1
-81.147	C#CCN(C)Cc1cc2cc(OCCCC3CCN(Cc4ccccc4)CC3)ccc2n1C
-80.540	CC(=O)c1ccc(OCCCCCCN2CCN(c3ncccn3)CC2)cc1O
-80.159	Cn1c(CNCc2ccccc2)cc2cc(OCCCC3CCN(Cc4ccccc4)CC3)ccc21
-79.860	CCN(CCCCCCOc1ccc(/C=C/c2cc(OC)cc(OC)c2)cc1)Cc1ccc(OC)cc1
-79.846	CCN(CCCCCCOc1cc(O)c2c(=O)c3ccccc3sc2c1)Cc1ccccc1OC
-79.827	Cn1c(CNCc2ccccc2)cc2cc(OCCCC3CCN(Cc4ccccc4)CC3)ccc21
-78.754	CN(CCCCCCOc1cc(O)c2c(=O)c3ccccc3sc2c1)Cc1ccccc1
-78.731	O=C1CCc2ccc(OCCCCCCN3CCC(N4CCCC4)CC3)cc2N1
-78.520	CCN(CC)CCCCCCOc1ccc(/C=C/c2cc(OC)cc(OC)c2)cc1
-78.326	CC(=O)c1ccc(OCCCCCN2CCN(c3ncccn3)CC2)cc1O
-78.296	C#CCNC(=O)c1cc2cc(OCCCC3CCN(Cc4ccccc4)CC3)ccc2n1C
-77.476	CCN(CCCCCCOc1ccc(/C=C/c2cc(OC)cc(OC)c2)cc1)Cc1ccccc1OC
-77.132	O=C1CCc2ccc(OCCCCN3CCN(c4ccccn4)CC3)cc2N1
-76.960	O=C1CCc2ccc(OCCCCCN3CCN(Cc4ccccc4)CC3)cc2N1
-76.925	Cn1c(CNc2ccccc2)cc2cc(OCCCC3CCN(Cc4ccccc4)CC3)ccc21
-76.135	CC(C)N1CCN(CCCCCCOc2ccc3c(c2)NC(=O)CC3)CC1
-75.651	COc1cc(/C=C/c2ccc(OCCCCCCN3CCOCC3)cc2)cc(OC)c1
-75.588	COc1cc2sn(CCCCCCN(C)Cc3ccccc3)c(=O)c2cc1OC
-75.335	O=c1cc(C(F)(F)F)c2ccc(OCCNC3CCN(Cc4ccccc4)CC3)cc2o1
-74.729	C#CCNCc1cc2cc(OCCCC3CCN(Cc4ccccc4)CC3)ccc2n1C
-74.632	CCN(CCCCOc1cc(O)c2c(=O)c3ccccc3sc2c1)Cc1ccccc1OC
-74.519	CCN(CCCCOc1cc(O)c2c(=O)c3ccccc3sc2c1)Cc1ccccc1
-74.426	O=C1CCc2ccc(OCCCCCN3CCN(c4ccccn4)CC3)cc2N1
-74.208	CCN(CCCCCCn1sc2cc(OC)c(OC)cc2c1=O)Cc1ccccc1
-73.901	C=CCNCc1cc2cc(OCCCC3CCN(Cc4ccccc4)CC3)ccc2n1C
-73.872	CCOc1cc(=O)oc2cc(OCCNC3CCN(Cc4ccccc4)CC3)ccc12
-73.720	O=c1oc2cc(OCCNC3CCN(Cc4ccccc4)CC3)ccc2c2c1CCCC2
-73.716	CCN(CCCCn1sc2cc(OC)c(OC)cc2c1=O)Cc1ccccc1OC
-73.652	CCN(CC)CCCCOc1ccc(/C=C2COc3ccccc3C2=O)cc1
-73.069	C#CCNCc1cc2cc(OCCCC3CCN(Cc4ccccc4)CC3)ccc2[nH]1
-72.774	CC(C)N1CCN(CCCCCN2C(=O)c3ccccc3C2=O)CC1
-72.583	C#CCN(C)[C@H](C)Cc1ccc(OCCCCCCCCNc2c3c(nc4cc(Cl)ccc24)CCCC3)cc1
-72.361	CC(=O)c1ccc(OCCCCN2CCN(c3ncccn3)CC2)cc1O

-72.221 COc1ccccc1CN(C)CCCCOc1cc(O)c2c(=O)c3ccccc3sc2c1
-72.188 CCN(CCCOc1cc(O)c2c(=O)c3ccccc3sc2c1)Cc1ccccc1OC
-72.119 CC(C)N1CCN(CCCCCOc2ccc3c(c2)NC(=O)CC3)CC1
-72.086 Cc1cc2ccc(OCCN3CCN(CCCNc4c5c(nc6ccccc46)CCCC5)CC3)cc2oc1=O
-71.807 COc1cccc2c(=O)cc(C(=O)NCCC3CCN(Cc4ccccc4)CC3)[nH]c12
-71.689 CCN(CCCCCCOc1ccc2c(C)c(C)c(=O)oc2c1)Cc1ccccc1
-71.377 COc1cc2c(cc1OC)C(=O)/C(=C/c1ccc(CN3CCCCC3)c(O)c1)CO2
-71.301 COc1ccccc1CN(C)CCCCCCOc1cc(O)c2c(=O)c3ccccc3sc2c1
-71.255 CCN(CCCCCCOc1ccc(/C=C/c2cc(OC)cc(OC)c2)cc1)Cc1ccccc1
-70.817 CC(=O)c1ccc(OCCCN2CCN(Cc3ccccc3)CC2)cc1O
-70.790 CC(=O)c1ccc(OCCCCCN2CCN(c3ccccn3)CC2)cc1O

Table S5: List of high affinity Top compounds as predicted from MM-GBSA set-2. The binding free energies are in kcal/mol

-97.498 C#CCN(C)Cc1cc2cc(OCCCC3CCN(Cc4ccccc4)CC3)ccc2n1C
-95.837 O=C1CCc2ccc(OCCCCCN3CCN(Cc4ccccc4)CC3)cc2N1
-94.364 CC(=O)c1ccc(OCCCCCCN2CCN(c3ncccn3)CC2)cc1O
-93.540 C#CCN(C)[C@H](C)Cc1ccc(OCCCCCCCCCNc2c3c(nc4ccccc24)CCCC3)cc1
-93.190 C#CCN(C)[C@H](C)Cc1ccc(OCCCCCCCCNc2c3c(nc4ccccc24)CCCC3)cc1
-93.038 CCOc1cc(=O)oc2cc(OCCNC3CCN(Cc4ccccc4)CC3)ccc12
-92.249 CCN(CCCCCCOc1ccc(/C=C/c2cc(OC)cc(OC)c2)cc1)Cc1ccc(OC)cc1
-92.235 C#CCN(C)[C@H](C)Cc1ccc(OCCCNc2c3c(nc4cc(Cl)ccc24)CCCC3)cc1
-91.406 CC(C)N1CCN(CCCCCN2C(=O)c3ccccc3C2=O)CC1
-90.575 O=C1CCc2ccc(OCCCCN3CCN(c4ccccn4)CC3)cc2N1
-88.720 C#CCN(C)[C@H](C)Cc1ccc(OCCCCCCCCCCNc2c3c(nc4ccccc24)CCCC3)cc1
-88.068 CN(CCCCCCOc1cc(O)c2c(=O)c3ccccc3sc2c1)Cc1ccccc1
-86.960 CC(C)N1CCN(CCCCOc2ccc3c(c2)NC(=O)CC3)CC1
-86.875 COc1cc2sn(CCCCCCN(C)Cc3ccccc3)c(=O)c2cc1OC
-86.821 Cn1c(CNc2ccccc2)cc2cc(OCCCC3CCN(Cc4ccccc4)CC3)ccc21
-86.757 CC(C)N1CCN(CCCCCCOc2ccc3c(c2)NC(=O)CC3)CC1
-86.518 O=c1oc2cc(OCCNC3CCN(Cc4ccccc4)CC3)ccc2c2c1CCCC2
-85.744 CCN(CCCCOc1cc(O)c2c(=O)c3ccccc3sc2c1)Cc1ccccc1OC
-85.648 CC(C)N1CCN(CCCOc2ccc3c(c2)NC(=O)CC3)CC1
-85.226 CC(C)N1CCN(CCCCCCN2C(=O)c3ccccc3C2=O)CC1
-84.977 CCN(CCCCCCOc1ccc2c(C)c(C)c(=O)oc2c1)Cc1ccccc1
-84.672 C#CCNCc1cc2cc(OCCCC3CCN(Cc4ccccc4)CC3)ccc2n1C
-84.601 O=c1cc(C(F)(F)F)c2ccc(OCCNC3CCN(Cc4ccccc4)CC3)cc2o1
-84.575 CCN(CCCCn1sc2cc(OC)c(OC)cc2c1=O)Cc1ccccc1OC
-84.270 CCN(CCCCCCn1sc2cc(OC)c(OC)cc2c1=O)Cc1ccccc1
-84.203 COc1cc2c(cc1OC)C(=O)/C(=C/c1ccc(CN3CCCCC3)c(O)c1)CO2
-84.126 CC(=O)c1ccc(OCCCCCCN2CCN(c3ccccn3)CC2)cc1O
-83.586 COc1cccc2c(=O)cc(C(=O)NCCC3CCN(Cc4ccccc4)CC3)[nH]c12
-83.031 C#CCN(C)Cc1cc2cc(OCCCN3CCCCC3)ccc2n1C
-82.899 CCN(CCCOc1cc(O)c2c(=O)c3ccccc3sc2c1)Cc1ccccc1OC
-82.860 C#CCN(C)[C@H](C)Cc1ccc(OCCCCCCCCNc2c3c(nc4cc(Cl)ccc24)CCCC3)cc1
-82.734 C=CCNCc1cc2cc(OCCCC3CCN(Cc4ccccc4)CC3)ccc2n1C
-82.240 CCN(CC)CCCCOc1ccc(/C=C2COc3ccccc3C2=O)cc1
-81.838 C#CCN(CCCOc1ccc2ccc(=O)oc2c1)Cc1ccccc1
-81.486 Cc1c(C)c2ccc(OCCNC3CCN(Cc4ccccc4)CC3)cc2oc1=O

-81.448	C#CCN(CCCCOc1ccc2ccc(=O)oc2c1)Cc1ccccc1
-81.181	Cc1c(C)c2ccc(OCCN3CCN(CCCNc4c5c(nc6ccccc46)CCCC5)CC3)cc2oc1=O
-80.654	COc1cc(/C=C/c2ccc(OCCN(C)Cc3ccccc3)cc2)cc(OC)c1
-80.553	COc1ccccc1CN(C)CCCCCCN1C(=O)c2ccccc2C1=O
-80.500	CCN(CCCCCCN1C(=O)c2ccccc2C1=O)Cc1ccccc1
-80.194	CN(CCCCOc1cc(O)c2c(=O)c3ccccc3sc2c1)Cc1ccccc1
-79.802	Cc1c(C)c2ccc(OCCCCCCN(C)Cc3ccccc3)cc2oc1=O
-79.779	O=C(NCCC1CCN(Cc2ccccc2)CC1)c1cc(=O)c2cc(O)ccc2o1
-79.497	CCOC(=O)c1cc2cc(OCCCC3CCN(Cc4ccccc4)CC3)ccc2[nH]1
-79.121	O=c1cc(C(F)(F)F)c2ccc(OCCN3CCN(CCCNc4c5c(nc6ccccc46)CCCC5)CC3)cc2o1
-78.671	COc1cc(/C=C/c2ccc(OCCCCCCN3CCC3)cc2)cc(OC)c1
-78.618	CC(C)N1CCN(CCCCN2C(=O)c3ccccc3C2=O)CC1
-78.597	O=c1oc2cc(OCCN3CCN(CCCNc4c5c(nc6ccccc46)CCCC5)CC3)ccc2c2c1CCCC2
-78.334	O=c1ccc2ccc(OCCCNC3CCN(Cc4ccccc4)CC3)cc2o1
-77.949	COc1cc2c(cc1OC)C(=O)C(CC1CCN(Cc3ccccc3)CC1)C2

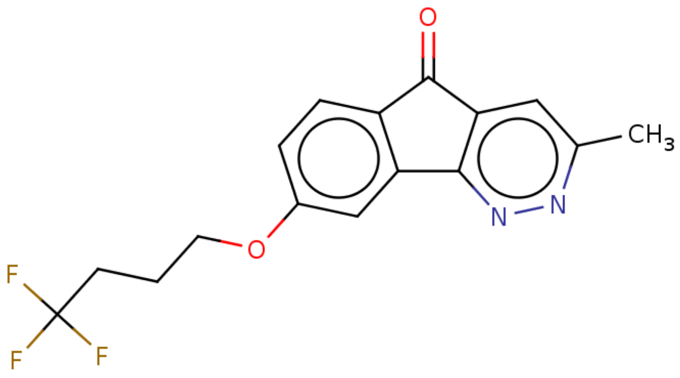

Figure S1: High affinity compound for MAO-B target as reported in experiments

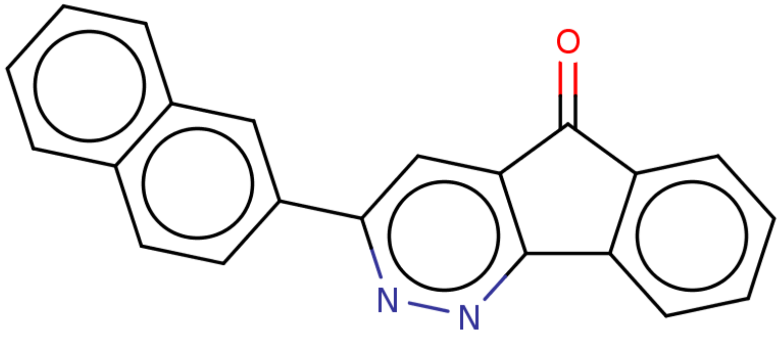

Figure S2: High affinity compound for MAO-B target as predicted from autodock-vina

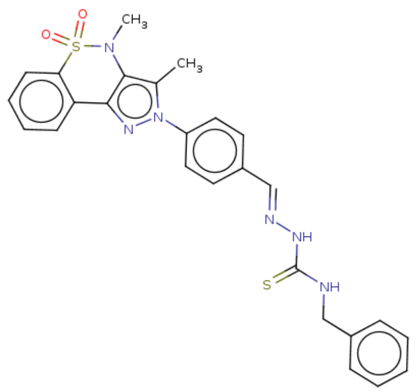

Figure S3: High affinity compound for MAO-B target as predicted from autodock4.0

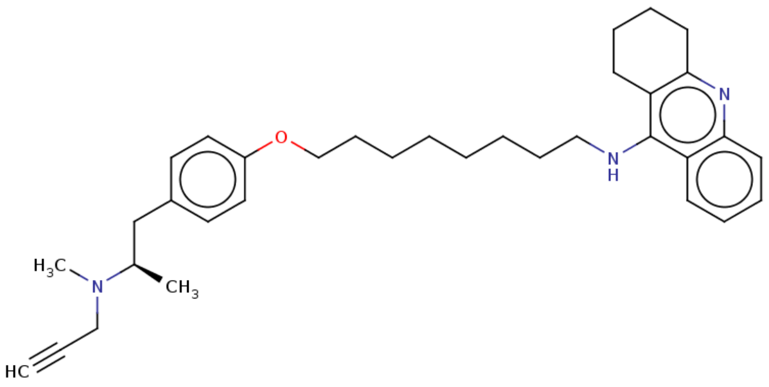

Figure S4: High affinity compound for MAO-B target as predicted from MM-GBSA set-1

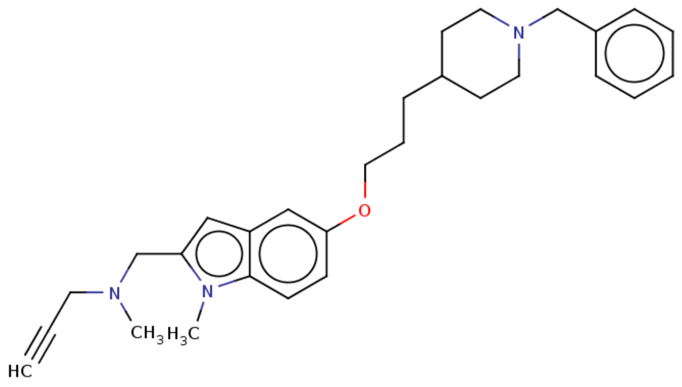

Figure S5: High affinity compound for MAO-B target as predicted from MM-GBSA set-2
